# Serum Soluble Tumor Necrosis Factor Receptors 1 and 2 Are Early Prognosis Markers After ST-Segment Elevation Myocardial Infarction

**DOI:** 10.3389/fphar.2021.656928

**Published:** 2021-09-01

**Authors:** Alexandre Paccalet, Claire Crola Da Silva, Laura Mechtouff, Camille Amaz, Yvonne Varillon, Charles de Bourguignon, Regine Cartier, Cyril Prieur, Danka Tomasevic, Nathalie Genot, Simon Leboube, François Derimay, Gilles Rioufol, Eric Bonnefoy-Cudraz, Nathan Mewton, Michel Ovize, Gabriel Bidaux, Thomas Bochaton

**Affiliations:** ^1^ INSERM U1060, CarMeN Laboratory, Groupement Hospitalier Est, Université de Lyon, Bron, France; ^2^ Stroke Department, Hôpital Wertheimer, Hospices Civils de Lyon, Bron, France; ^3^ Centre D’investigation Clinique de Lyon, Hôpital Louis Pradel, Hospices Civils de Lyon, Bron, France; ^4^ Centre de Biologie Est, Groupement Hospitalier Est, Hospices Civils de Lyon, Bron, France; ^5^ Unité de Soins Intensifs Cardiologiques, Hôpital Louis Pradel et Université Claude Bernard, Hospices Civils de Lyon, Bron, France; ^6^ Department of Interventional Cardiology, Cardiovascular Hospital and Claude-Bernard University, Bron, France; ^7^ Service D’explorations Fonctionnelles Cardiovasculaires, Hôpital Louis Pradel, Hospices Civils de Lyon, Bron, France

**Keywords:** myocardial infarction, TNF, soluble TNF receptor, biomarker, prognosis

## Abstract

**Background:** As inflammation following ST-segment elevation myocardial infarction (STEMI) is both beneficial and deleterious, there is a need to find new biomarkers of STEMI severity.

**Objective:** We hypothesized that the circulating concentration of the soluble tumor necrosis factor α receptors 1 and 2 (sTNFR1 and sTNFR2) might predict clinical outcomes in STEMI patients.

**Methods:** We enrolled into a prospective cohort 251 consecutive STEMI patients referred to our hospital for percutaneous coronary intervention revascularization. Blood samples were collected at five time points: admission and 4, 24, 48 h, and 1 month after admission to assess sTNFR1 and sTNFR2 serum concentrations. Patients underwent cardiac magnetic resonance imaging at 1 month.

**Results:** sTNFR1 concentration increased at 24 h with a median of 580.5 pg/ml [95% confidence interval (CI): 534.4–645.6]. sTNFR2 increased at 48 h with a median of 2,244.0 pg/ml [95% CI: 2090.0–2,399.0]. Both sTNFR1 and sTNFR2 peak levels were correlated with infarct size and left ventricular end-diastolic volume and inversely correlated with left ventricular ejection fraction. Patients with sTNFR1 or sTNFR2 concentration above the median value were more likely to experience an adverse clinical event within 24 months after STEMI [hazards ratio (HR): 8.8, 95% CI: 4.2–18.6, *p* < 0.0001 for sTNFR1; HR: 6.1, 95% CI: 2.5 –10.5, *p* = 0.0003 for sTNFR2]. Soluble TNFR1 was an independent predictor of major adverse cardiovascular events and was more powerful than troponin I (*p* = 0.04 as compared to the troponin AUC).

**Conclusion:** The circulating sTNFR1 and sTNFR2 are inflammatory markers of morphological and functional injury after STEMI. sTNFR1 appears as an early independent predictor of clinical outcomes in STEMI patients.

## Introduction

Despite major progress during the past two decades, the mortality and morbidity of patients with ST-segment elevation myocardial infarction (STEMI) remain too high. Recent data indicate that, despite optimal treatment, cardiovascular mortality at 1 year was 6.8% and hospitalization rate for heart failure was 22.8% in anterior STEMI (Cung et al., 2015). While most patients display limited or no residual cardiac functional alteration as a consequence of myocardial infarction, some develop heart failure, and they have a poor prognosis. An early evaluation of a potential adverse clinical outcome would certainly help personalize the prevention of such a detrimental evolution. For this, infarct size, as measured by cardiac magnetic resonance imaging or even cardiac enzyme release, is a good indicator of myocardial damage and a fair prognostic factor; it is however not measured in daily clinical practice. Left ventricular ejection fraction (LVEF) is also a valid estimate of prognosis, but the variability and operator dependence of its assessment by echocardiography are worrisome. We therefore lack an early, easy-to-use, and reliable biomarker to distinguish between patients with a good and a poor prognosis and to personalize treatment and follow-up.

Tumor necrosis factor alpha (TNF-α), which is involved in several pathologies such as septic shock and rheumatoid arthritis (Spooner et al., 1992; Yamanaka, 2015), has pleiotropic effects and regulates the expression of several inflammatory genes (Vilcek and Lee, 1991). Cardiac cells, particularly cardiomyocytes, secrete TNF-α in response to endotoxin stimuli (Kapadia et al., 1995) or ischemic stress (Belosjorow et al., 1999; Irwin et al., 1999), and it plays a central role in cardiovascular diseases, specifically in atherothrombosis and ischemia–reperfusion injury (Levine et al., 1990; Munkvad et al., 1991; Kurrelmeyer et al., 2000; Flaherty et al., 2008)*.* The TNF-α pathway has also been involved in heart failure, although inhibition of TNF-α (Levine et al., 1990; Torre-Amione et al., 1996) in heart failure patients was not associated with clinically relevant benefit (Shetelig et al., 2018). It is of note that a major increase of TNF-α activity induces myocardial and cardiomyocyte dysfunction essentially through TNFR1, and low overexpression of TNF-α in the mouse heart increases contractile performance via TNFR1. TNF-α signaling is also implicated in cardiac remodeling, and inhibiting TNF-α in the acute phase of Myocardial Infarction (MI) promotes ventricular rupture by reducing fibrosis via the activation of matrix metalloproteinase-9 (Monden et al., 2007).

One might therefore question whether circulating TNF-α could be a prognosis marker in STEMI patients. Unfortunately, TNF-α is not stable after blood sampling (Friebe and Volk, 2008); however, since TNF-α induces the release of soluble TNF receptors (sTNFRs) into the circulation (Lantz et al., 1990), the measurement of sTNFR allows to indirectly evaluate TNF-α activity. Two main TNF-α receptors exist: type 1 (TNFR1) and type 2 (TNFR2) (Munkvad et al., 1991). Both contain transmembrane domains but can also be cleaved by ADAM metallopeptidase domain 17 into soluble receptors (sTNFR1 and sTNFR2). TNFR1 is ubiquitously expressed and is considered a death receptor due to its role in apoptosis, whereas TNFR2 is mainly expressed in T-cells and stimulates NFκB signaling (Wajant and Siegmund, 2019). Soluble TNFR1 (sTNFR1) and soluble TNFR2 (sTNFR2) compete with the transmembrane forms by binding circulating TNF-α and thereby inhibiting its action. TNFR1 is classically considered pro-inflammatory, whereas TNFR2 has been associated with anti-inflammatory functions (Faustman and Davis, 2010).

We hypothesized that recording the early kinetics of soluble TNFR1 and TNFR2 in STEMI patients would reflect the severity of the inflammatory response to a prolonged ischemia–reperfusion insult and might be used to evaluate the prognosis of STEMI patients. Specifically, we assessed the association of sTNFR with surrogates of cardiac damage such as infarct size, LVEF, or adverse LV remodeling as well as with clinical outcomes.

## Methods

### Study Design

The study cohort (HIBISCUS cohort: coHort of patients to Identify Biological and Imaging markerS of CardiovascUlar outcomes in ST elevation myocardial infarction) was composed of consecutive patients admitted to our institution (a tertiary referral university hospital) for a suspected STEMI from 2016 to 2019. The study was approved by our institutional review board and ethics committee and is in accordance with the Declaration of Helsinki principles. All patients gave written informed consent. STEMI was defined according to the European Society of Cardiology guidelines by the presence of clinical symptoms (chest pain) associated with an ST elevation of more than 2 mm in two contiguous leads on a standard 12-lead electrocardiogram and significant troponin I elevation. All patients underwent coronary angiography at admission with subsequent reperfusion by primary percutaneous intervention. All patients had a complete myocardial enzyme release assessment and underwent contrast-enhanced cardiac magnetic resonance imaging at 1 month after AMI for measurement of infarct size and LV function.

All individual clinical, treatment, and outcome data were stored prospectively in the database of the center for clinical investigation of Hospices Civils de Lyon. Adverse clinical events were registered at follow-up visits scheduled at 1 month, 1 year, and 2 years after index hospitalization. The primary endpoint was defined as the composite of all-cause death, rehospitalization for heart failure, recurrent infarction, and stroke before any analysis, as previously described (Piot et al., 2008). The ClinicalTrials.gov identifier is NCT03070496.

For the present analysis, 251 patients with at least one-year clinical follow-up in the study cohort were included.

### Blood Sample Collection

Sera from all patients were collected at five time points: admission and 4 h (4 h), 24 h (24 h), 48 h (48 h), and 1 month after PCI revascularization. Samples were frozen at −80°C and stored at the local hospital biobank until assay (NeuroBioTec Biological Resource Centre). All sera from the study population were thawed only once to avoid biomarker alteration.

### Biomarker Measurement

Circulating sTNFR1 and sTNFR2 concentrations were assessed using ELISA (R&D Systems ELISA Kit, Minneapolis, MN, United States). The sensitivity of R&D Kit was calculated using the mean value of multiple blank measurements (reagent diluent only). The minimum detectable concentration was defined as 2 SD above this mean; it was 2.1 pg/ml for sTNFR1 and 74.5 pg/ml for sTNFR2.

C-reactive protein (CRP) was assessed using immunoturbidimetry (Abbott Laboratories, Chicago, IL, United States). Highly sensitive troponin I (immuno-chemiluminescence, Abbott Laboratories, Chicago, IL, United States) and total creatine kinase levels (Beckman Coulter, Inc.; expressed in IU/L) were measured at admission and 4, 24, and 48 h after PCI. Leukocytes (neutrophil granulocytes, monocytes, and lymphocytes) were collected at admission and 24 h, 48 h, and 1 month after admission and assessed using fluorescence-activated cell sorting (XN-9000; Sysmex, Kobe, Japan).

### Cardiac Magnetic Resonance Analysis

Patients underwent cardiac magnetic resonance imaging at 1 month and 1 year after STEMI. All patients were scanned in a supine position on a 1.5 T MAGNETOM Avanto TIM system (Siemens Healthineers, Erlangen, Germany), as previously described (Bernelin et al., 2019). Cine free precession sequences in two-chamber, four-chamber, and ventricular short-axis planes were used for quantitative ventricular measurements. Myocardial delayed enhancement sequences were assessed in short- and long-axis planes with a non-selective 180° inversion recovery 10–15 min after the administration of 0.2 mmol/kg gadolinium-based contrast agent. Infarct size was measured using the CMRSegTools segmentation plugin (CREATIS, Lyon, France) with OsiriX software (Pixmeo, Geneva, Switzerland). Late gadolinium enhancement regions were quantified with a full width at half maximum algorithm, and infarct size was expressed as a percentage of the left ventricular mass. LVEF, left ventricular end-diastolic volume index, left ventricular end-systolic volume index, and left ventricular mass were measured offline on all short-axis views in the cine images (Philips View Forum; Philips Healthcare, Amsterdam, the Netherlands).

### Statistical Analysis

Data are expressed as median and 95% confidence interval (CI) or interquartile range (IQR) or mean and standard deviation according to their distribution. Categorical data are expressed in number and proportion (%). Comparisons between the different time points and the admission time were performed using the Kruskal–Wallis test. A paired *t*-test was used for continuous variables with parametric distribution. Receiver-operating characteristic (ROC) curves were compared using DeLong *et al.*’s test (DeLong et al., 1988). Correlations were tested using Spearman correlation because of their non-parametric distribution. Multivariate analysis was performed using Cox proportional-hazards regression. This model was adjusted on the variables associated with the major adverse cardiac event occurrence after STEMI based on the results of a previous study (Bochaton et al., 2019): age, sex, diabetic status, hypertension, smoking status, renal dysfunction, and troponin peak. Statistical analyses were performed using GraphPad Prism 8.01 (San Diego, California, United States), with the exception of DeLong et al.’s test and multivariate analysis that were performed using MedCalc Version 12.4.0.0 (Ostend, Belgium).

## Results

### Study Population

Characteristics of the study population are presented in Table 1. Briefly, 251 patients were included; the mean ± SD of age was 59 ± 12 years, and 79.3% were male. There were 132 anterior infarcts (52.6%); 167 patients (66.5%) had TIMI flow grade 0–1 before, and 230 (91.6%) had thrombolysis in myocardial infarction (TIMI) flow grade 2–3 after primary percutaneous intervention. Two hundred sixteen patients (86.1%) were of Killip class 1 at admission. Peak CRP occurred at 48 h, and its median (IQR) value was 18.1 mg/L (7.6–52.0), and the median (IQR) leukocyte count was 11.8 G/L (9.3–14.5) at admission.

**TABLE 1:** Characteristics of the study population.

	All patients (*n* = 251)
Age, years	59 ± 12
Male, N (%)	199 (79.3)
Body mass index, kg/m^2^	26.3 (23.8–29.4)
Hypertension, N (%)	70 (27.9)
Hypercholesterolemia, N (%)	70 (27.9)
Diabetes mellitus, N (%)	37 (14.7)
Current smoker, N (%)	126 (50.2)
Time from symptoms to primary percutaneous intervention, min	200 (145–315)
Anterior myocardial infarction, N (%)	132 (52.6)
Killip status = 1, N (%)	216 (86.1)
TIMI flow grade at admission = 0–1	167 (66.5)
Post-primary percutaneous intervention TIMI flow grade >2 (%)	230 (91.6)
LVEF at baseline (%)	55.0 (46.0–61.0)
eGFR <60 (mL/min/1.73m^2^)^a^	13 (5.2)
Peak troponin I, ng/L	43,904 (15,731–114,083)
Peak creatine kinase, mUI/L	1,561 (686–3,666)
Peak CRP, mg/L	17.9 (7.2–47.2)
Leucocyte count at admission, G/L	11.8 (9.3–14.5)

TIMI, thrombolysis in myocardial infarction; LVEF, left ventricular ejection fraction; CRP, C-reactive protein.

a eGFR: estimated glomerular filtration rate using the Chronic Kidney Disease Epidemiology Collaboration (CKD-EPI) formula corrected for race.

### Kinetics of sTNFR1 and sTNFR2 Following STEMI

**FIGURE 1:**
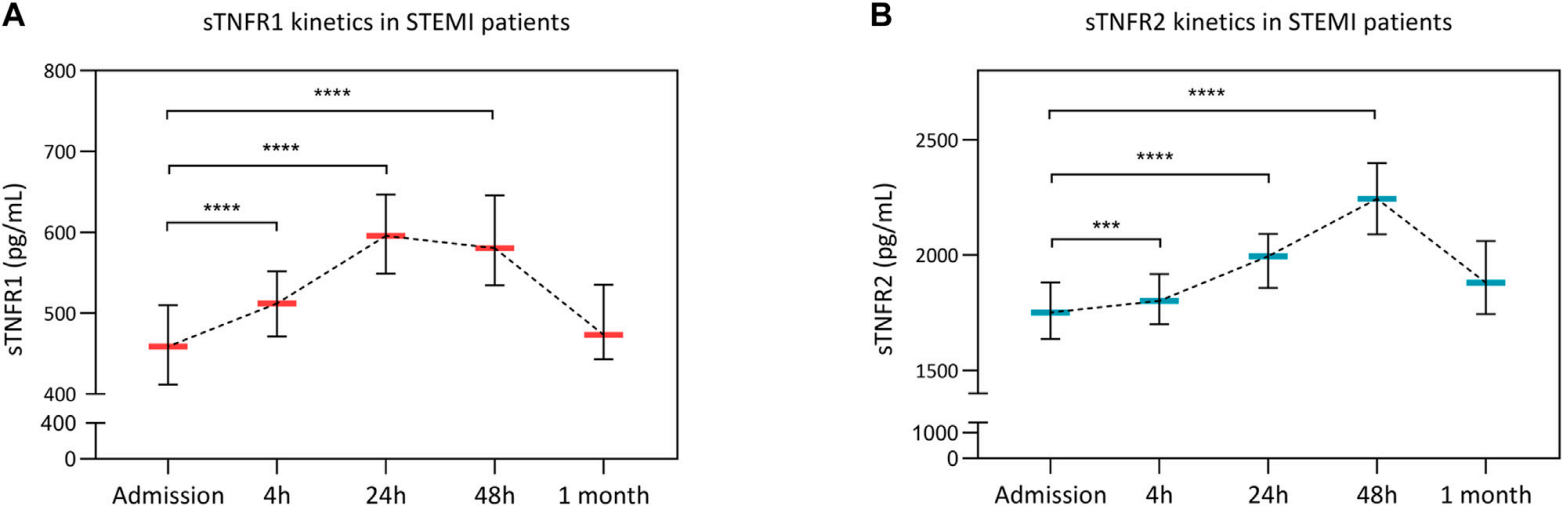
Soluble tumor necrosis factor receptor 1 (sTNFR1) and sTNFR2 kinetics after STEMI. Circulating concentrations of sTNFR1 **(A)** and sTNFR2 **(B)** at admission and 4, 12, 48 h, and 1 month after PCI revascularization are presented. Time points were compared using the Kruskal–Wallis test. **p < 0.01, ****p < 0.0001 versus admission.

Temporal variations of circulating sTNFR1 and sTNFR2 are presented in Figure 1. Blood sTNFR1 and sTNFR2 significantly increased over the first 48 h after acute myocardial infarction. sTNFR1 reached a peak at 24 h, and the median value was 580.5 pg/ml [95% CI: 534.4–645.6]. The sTNFR2 peak occurred at 48 h, and the median value was 2,244.0 pg/ml [95% CI: 2090.0–2,399.0]. At 1 month, sTNFR1 and sTNFR2 values both returned to near baseline values. Troponin I, C-reactive protein (CRP), and creatine kinase (CK) kinetics are presented in Supplementary Figure 1.

### Soluble TNFRs, Infarct Size, LVEF, and Left Ventricle Remodeling

The median (IQR) cardiac magnetic resonance imaging infarct size was 14.4% (6.9–24.5) of left ventricle (LV) mass. The median (IQR) admission LVEF was 55.0% (46.0–61.0), and the median (IQR) indexed left ventricular end-diastolic volume was 87.6 ml/m^2^ (75.3–96.2). Both 48 h sTNFR1 and sTNFR2 were significantly, but weakly correlated with Infarct size (IS) (r = 0.17, *p* = 0.03, and r = 0.20, *p* = 0.01, respectively) and inversely correlated with LVEF (r = -0.15, *p* = 0.047, and r = -0.18, *p* = 0.01, respectively). Furthermore, 48 h sTNFR1 and sTNFR2 levels were significantly correlated with cardiac remodeling evaluated on the indexed left ventricular end-diastolic volume and left ventricular end-systolic volume (r = 0.16, *p* = 0.04, and r = 0.19, *p* = 0.02, respectively). Cardiac remodeling data are presented in Table 2.

**TABLE 2:** Cardiac remodeling data according to sTNFR levels.

	sTNFR1 ≤ median	sTNFR1 > median	p value
LVEF at 1 month (%)	54.0 (46.0–59.0)	50.5 (43.3–58.0)	0.19
LVEDV at 1 month (ml)	170.0 (136.8–199.5)	171.0 (145.0–191.0)	0.96
LVESV at 1 month (ml)	80.5 (62.8–100.0)	85.0 (64.0–100.0)	0.87
	**sTNFR2 ≤ median**	**sTNFR2 > median**	**p value**
LVEF at 1 month (%)	56.0 (47.3–59.0)	50.0 (44.0–56.0)	0.02
LVEDV at 1 month (ml)	162.5 (136.8–185.3)	181.0 (147.0–198.0)	0.045
LVESV at 1 month (ml)	79.0 (57.8–96.8)	87.0 (67.0–108.0)	0.05

LVEF, left ventricular ejection fraction; LVEDV, left ventricular end-diastolic volume; LVESV, left ventricular end-systolic volume. Comparison between groups was performed using the Mann–Whitney test.

### Circulating Soluble TNF Receptors and Clinical Outcomes

Thirty-two patients (12.7% of study population) experienced at least one major adverse cardiac–cerebral event during the 24-month follow-up (5 deaths, 7 myocardial infarction, 16 hospitalizations for heart failure, and 4 strokes). Among these 32 patients, 48 h serum was available for 29 of them.

**FIGURE 2:**
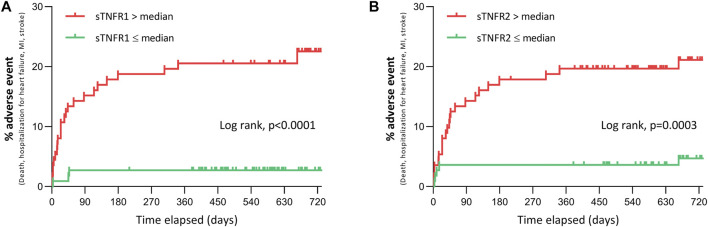
Adverse clinical events at 24 months after STEMI according to 48 h sTNFR1 and sTNFR2. **(A, B)** Adverse clinical events (all-cause mortality, hospitalization for heart failure, myocardial infarction, or stroke) over 24 months after admission for suspected STEMI in patients with sTNFR1 **(A)** or sTNFR2 **(B)** concentration at 48 h either below or above the median value of the study population.

As presented in Figure 2, patients with high circulating 48 h sTNFR1 or sTNFR2 (above the median value) were more likely to undergo the composite event of death, recurrent myocardial infarction, or stroke, as well as hospitalization for heart failure over the 24-month follow-up; the respective unadjusted hazards ratio (HR) was 8.8, with 95% CI of 4.2–18.6 and *p* < 0.0001, and 5.0, with 95% CI of 2.4–10.5 and *p* = 0.0003. In the group with sTNFR1 below the median value, five patients experienced adverse cardiac events, three hospitalizations for heart failure, and two MI. In the group with the sTNFR1 level above the median value, 24 patients experienced adverse cardiac outcomes, 2 strokes, 5 deaths, 6 myocardial infarction, and 11 hospitalizations for heart failure. In the group with sTNFR2 below the median value, four patients experienced adverse cardiac events, three hospitalizations for heart failure, and one myocardial infarction. In the group with sTNFR2 above the median value, 25 patients experienced adverse cardiac outcomes, 11 hospitalizations for heart failure, 7 myocardial infarction, 2 strokes, and 5 deaths.

Stratifying the sTNFR1 and sTNFR2 values by quartiles found that the risk was significantly different between groups (log-rank: *p* < 0.0001 for sTNFR1 and sTNFR2) and that this increased in the function of quartile (Supplementary Figure 2).

**FIGURE 3:**
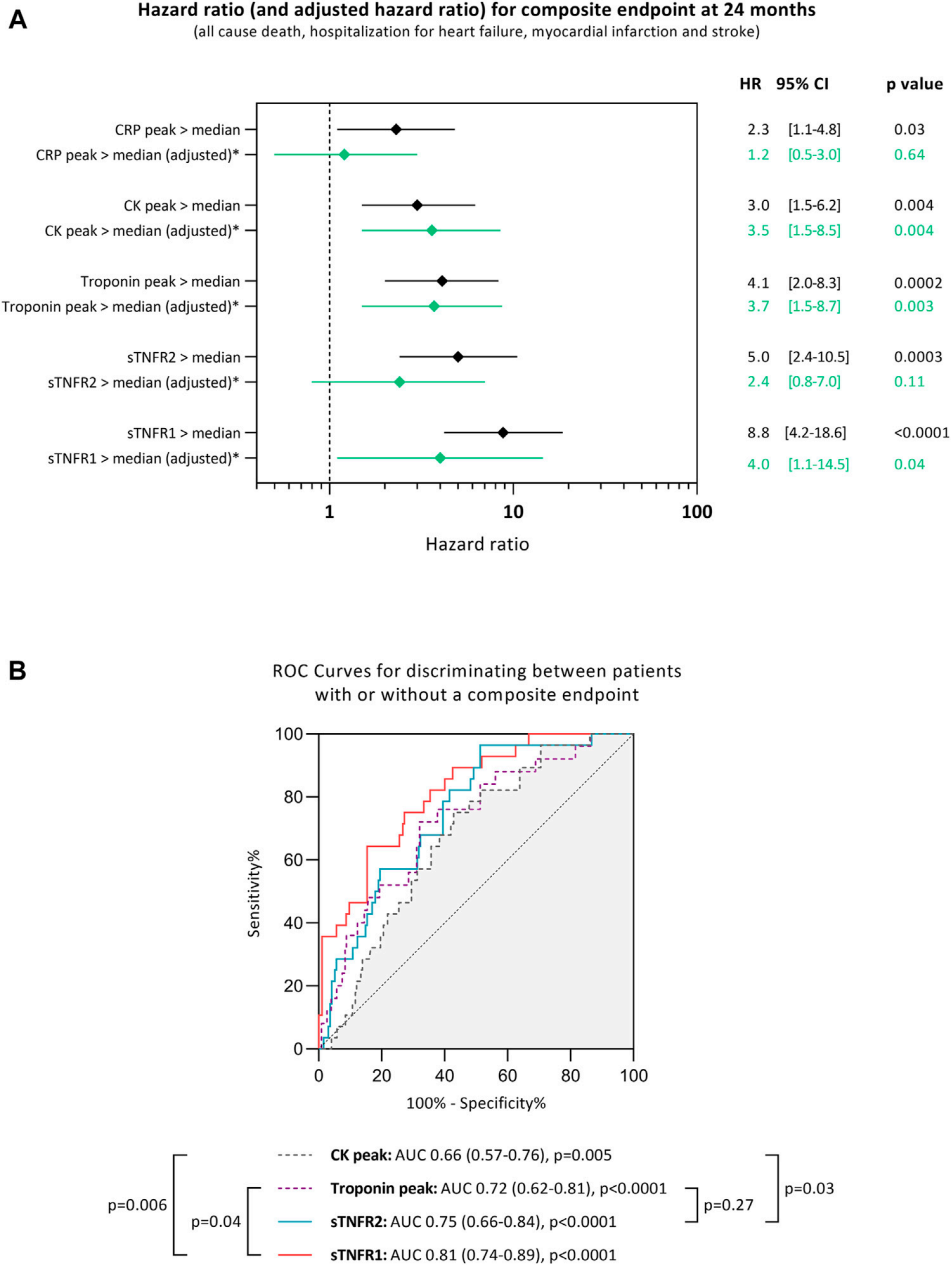
Unadjusted hazards ratio (HR) and 95% confidence interval (CI) for experiencing a composite endpoint during the median of 24 months of follow-up when having high soluble tumor necrosis factor receptor (sTNFR) 1 or high sTNFR2 (>median), high C-reactive protein (CRP > median), high troponin peak (>median), and high creatine kinase (CK) peak (>median). The peak used is the maximum value of troponin, creatine kinase, or CRP measured for each patient individually **(A)**. Receiver-operating characteristic (ROC) curves for discriminating between patients with or without a cardiac adverse event during the 24 months of follow-up after STEMI according to different markers **(B)**. AUC: area under the curve, sTNFR1: soluble tumor necrosis factor receptor 1, and sTNFR2: soluble tumor necrosis factor receptor 2. *Hazards ratio adjusted in multivariable Cox regression analyses with models including age, sex, diabetes, hypertension, smoking, renal dysfunction, troponin peak, TIMI flow grade after PCI, and high levels of sTNFR1 (>median) or high levels of sTNFR2 (>median).

In multivariable Cox regression analyses with models including age, sex, diabetes, hypertension, smoking status, renal dysfunction (eGFR <60 ml/min/1.73 m^2^), troponin I peak, TIMI flow grade after primary percutaneous intervention, and high 48 h levels of sTNFR1 or high 48 h levels of sTNFR2, sTNFR1 was associated with an increased risk of experiencing the composite endpoint during the 24 months of follow-up [adjusted HR: 4.1, 95% CI: 1.1–15.0, *p* = 0.03], whereas sTNFR2 was not [adjusted HR: 2.1, 95% CI: 0.7–6.2, *p* = 0.18; Figure 3A].

The ability of sTNFR1 and sTNFR2 to discriminate between patients experiencing the composite adverse event was assessed using ROC curves. Soluble TNFR1 had an AUC of 0.81 [95% CI: 0.74–0.89, *p* < 0.0001], while sTNFR2 had an AUC of 0.75 [95% CI: 0.66–0.84, *p* < 0.0001]. Both sTNFR1 and sTNFR2 could better discriminate patients experiencing the composite adverse event when compared to peak CK (*p* = 0.006 and *p* = 0.03, respectively, compared to the CK AUC). Only sTNFR1 was independently associated with the occurrence of the composite adverse event when compared to troponin I (*p* = 0.04 compared to the troponin AUC; Figure 3B).

## Discussion

A major finding of the present study is that early kinetics of circulating sTNFR1 and sTNFR2 bring new important information as to the prognosis of STEMI patients.

To our knowledge, this is the first study to assess the kinetics of sTNFR1 and sTNFR2 during the first hours following acute myocardial infarction and up to 1 month; there were a peak at 24 h for sTNFR1 and at 48 h for sTNFR2 and a return to near initial values at 1 month. This 24-hour to 48-hour delay might rather correspond to the inflammatory response associated with the cardiac healing/remodeling process rather than to the myocardial damage per se. Indeed, sTNFR1 is expressed in many cell types, whereas sTNFR2 seems to be only expressed in cardiac cells and in immune cells (Urschel and Cicha, 2015). To evaluate the cardiac-specific origin, coronary sinus circulation should have been collected as performed in another study (Pérez-Martínez et al., 2018). This interpretation also is supported by the better correlation herein of sTNFR with LVEF changes than with infarct size. The low variation of LVEF and IS values compared to the high variation of sTNFR value could explain the weak correlation. The results are, however, in contradiction with a previous study reported by Valgimigli *et al.* that suggests that admission (i.e., pre-PCI) values of sTNFR1, but not sTNFR2, were predictive of clinical outcomes (Valgimigli et al., 2005). This apparent discrepancy might be due to, first, the recruitment by the authors of both ST-segment elevated and non-ST-segment elevated MI and, second, the measurement of sTNFR at a single time point (i.e., admission).

Soluble and membrane-bound TNFR1 and TNFR2 represent a highly regulated system playing a central role in cell death and inflammatory response (Naudé et al., 2011). Levine *et al.* first reported that serum TNF-α is increased in patients with severe heart failure (Levine et al., 1990), and TNF-α antagonists have been assessed in patients with heart failure. TNF-α antagonists include a recombinant human TNF receptor (etarnecept) that binds and inhibits the circulating TNF-α and a chimeric IgG antibody (infliximab) that binds both the soluble and transmembrane TNF-α. Unfortunately, multicenter randomized trials (RENEWAL study for etanercept and ATTACH trial for infliximab) (Chung et al., 2003; Mann et al., 2004) failed to demonstrate any significant improvement of heart failure. We demonstrated here that patients with high circulating sTNFR1 or sTNFR2 were at high risk of adverse clinical events, among which heart failure was predominant. Circulating sTNFR1 or sTNFR2 was independent of cardiac enzyme release and brought additional information as to the patients’ prognosis. The sTNFR1 level at 48 h could, even better than troponin, discriminate patients experiencing a composite event. Although sTNFR1 is an independent prognosis biomarker, it is influenced by age, sex, diabetic status, smoking, renal dysfunction, and infarct size assessed by the troponin peak. While the potential of sTNFR1 and sTNFR2 as targets for drug development remains to be determined, this study strongly suggests that they might be used as prognosis markers.

### Study Limitations

The size of the study population is relatively small, but, to date, it is the largest investigation of sTNFR levels after STEMI. Furthermore, we were not able to measure TNF-α in the serum of our patient probably due to its short half-life in the sera. Finally, the follow-up is relatively short (18 months), but it was enough to show a significant difference in terms of clinical outcomes, and the number and type of clinical events after STEMI are in line with those in previous studies (Shetelig et al., 2018).

## Conclusion

In STEMI patients, circulating sTNFR1 and sTNFR2 significantly increased within 2 days after PCI. This increase was significantly associated with the clinical outcome. sTNFR might bring additional information and help personalize STEMI patients’ follow-up and care.

## Data Availability

The raw data supporting the conclusions of this article will be made available by the authors, upon request.
