# Supplementary material for: Serum Soluble Tumor Necrosis Factor Receptors 1 and 2 Are Early Prognosis Markers After ST-Segment Elevation Myocardial Infarction
Source: Front Pharmacol. 2021 Sep 1;12:656928. doi: 10.3389/fphar.2021.656928 (PMC8440863; doi:10.3389/fphar.2021.656928)
Supplement: Supplementary file 1 [file Image2.pdf]

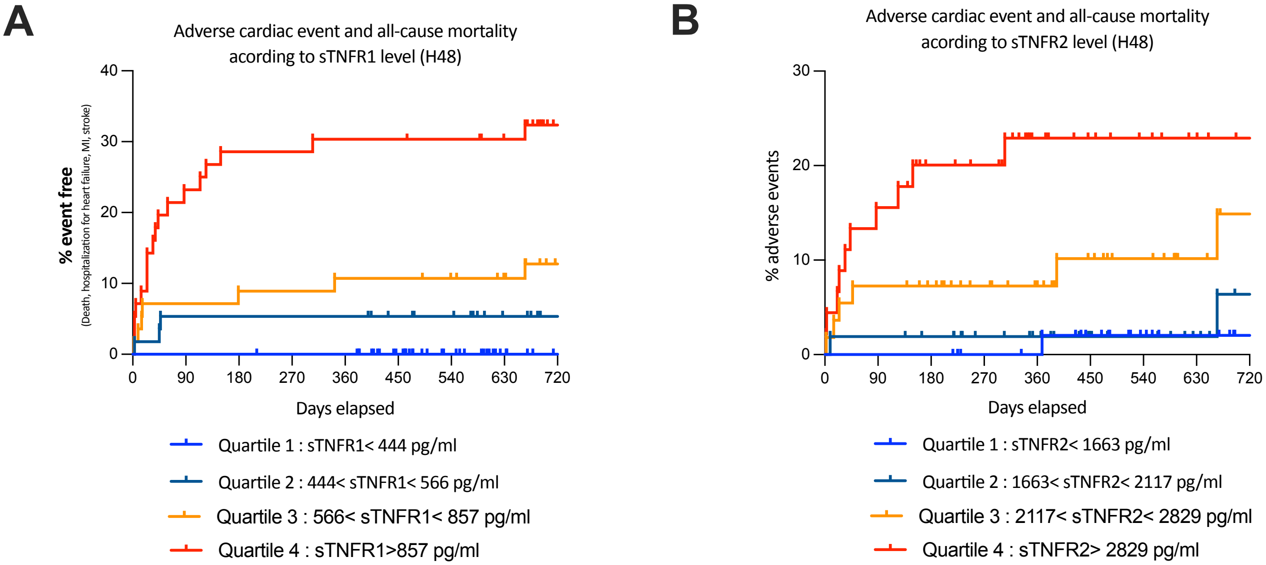
A
Adverse cardiac event and all-cause mortality
acording to sTNFR1 level (H48)
% event free
(Death, hospitalization for heart failure, MI, stroke)
40
30
20
10
0
0
90
180
270
360
450
540
630
720
Days elapsed
Quartile 1 : sTNFR1< 444 pg/ml
Quartile 2 : 444< sTNFR1< 566 pg/ml
Quartile 3 : 566< sTNFR1< 857 pg/ml
Quartile 4 : sTNFR1>857 pg/ml
B
Adverse cardiac event and all-cause mortality
acording to sTNFR2 level (H48)
% adverse events
30
20
10
0
0
90
180
270
360
450
540
630
720
Days elapsed
Quartile 1 : sTNFR2< 1663 pg/ml
Quartile 2 : 1663< sTNFR2< 2117 pg/ml
Quartile 3 : 2117< sTNFR2< 2829 pg/ml
Quartile 4 : sTNFR2> 2829 pg/ml
