# Supplementary material for: Serum Soluble Tumor Necrosis Factor Receptors 1 and 2 Are Early Prognosis Markers After ST-Segment Elevation Myocardial Infarction
Source: Front Pharmacol. 2021 Sep 1;12:656928. doi: 10.3389/fphar.2021.656928 (PMC8440863; doi:10.3389/fphar.2021.656928)
Supplement: Supplementary file 2 [file Image1.pdf]

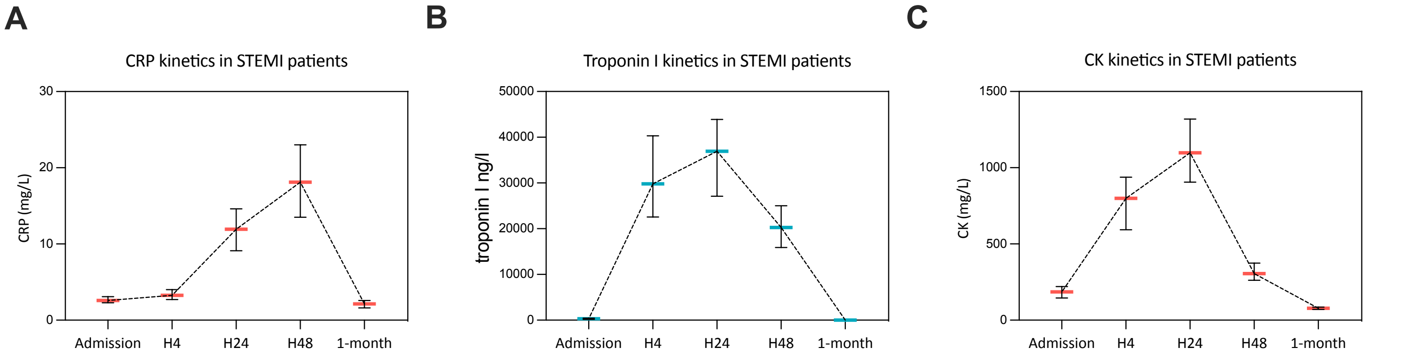
A
CRP kinetics in STEMI patients
CRP (mg/L)
30
20
10
0
Admission
H4
H24
H48
1-month
B
Troponin I kinetics in STEMI patients
troponin I ng/l
50000
40000
30000
20000
10000
0
Admission
H4
H24
H48
1-month
C
CK kinetics in STEMI patients
CK (mg/L)
1500
1000
500
0
Admission
H4
H24
H48
1-month
